# Upadacitinib for Spondyloarthritis; A new treatment option

**DOI:** 10.1016/j.amsu.2022.104664

**Published:** 2022-09-16

**Authors:** Manahil Shekha, Omer Ahmed Shaikh, Gulrukh Shaikh, Sidhant Ochani, Kaleem Ullah

**Affiliations:** a Department of Medicine, Ziauddin Medical University, Karachi, Pakistan; b Department of Medicine, Liaquat National Hospital and Medical College, Karachi, Pakistan; c Department of Medicine, Khairpur Medical College, Khairpur Mir's, Pakistan; d Department of Liver Transplantation, Pir Abdul Qadir Shah Institute of Medical Sciences, Ghambat, Pakistan

## Abstract

Axial spondyloarthritis, often known as ankylosing spondylitis (AS), is an inflammatory condition that mostly affects the axial skeleton. Axial spondyloarthritis is further subdivided into non-radiographic and radiographic AS. For radiographic axial spondyloarthritis, the male-to-female ratio is two to one, while for non-radiographic axial spondyloarthritis, it is one to one, often manifesting in the third decade of life. Effective treatment for AS includes non-steroidal anti-inflammatory medications (NSAIDs) and TNF blockers. All articular symptoms of AS have been seen to improve significantly when treated with TNF inhibitors such as Etanercept, Adalimumab, Infliximab, Certolizumab, and Golimumab. Upadacitinib, has proven to be significantly efficacious in the management of active non-radiographic axial spondyloarthritis (nr-axSpA), with MRI-based or blood tests displaying objective evidence of inflammation, an increased C-reactive protein, and an unsatisfactory response to Non-steroidal anti-inflammatory drug (NSAIDs). the lack of oral therapy options, and the stigma associated with surgical intervention makes it crucial to offer an unambiguous treatment choice, especially in light of the disease's strong heredity. Thus, Upadacitinib's usage in the treatment of nr-axSpA and its clinical trial is a significant step toward the availability of an internationally-approved medicine for the treatment of nr-axSpA.

Respected Editor,

Axial spondyloarthritis, often known as ankylosing spondylitis (AS), is an inflammatory condition that mostly affects the axial skeleton. Axial spondyloarthritis is further subdivided into non-radiographic and radiographic AS. For radiographic axial spondyloarthritis, the male-to-female ratio is two to one, while for non-radiographic axial spondyloarthritis, it is one to one, often manifesting in the third decade of life [1]. The prevalence of AS in the general population ranges from 9 to 30 per 10,000 people worldwide [2]. Patients often present with persistent lower back, pelvic pain, and stiffness, however, any portion throughout the spine may be affected [1].

The estimated heritability is greater than 90%, with the strongest genetic link being HLA-B27. According to data from clinical studies, Interleukin-17 (IL-17) and Tumor necrosis factor (TNF)-α also appear to play a significant role in its pathogenesis [1].

Effective treatment for AS includes non-steroidal anti-inflammatory medications (NSAIDs) and TNF blockers [1]. Early use of NSAIDs, together with physical therapy and exercise, is an imperative part of this treatment strategy [2]. All articular symptoms of AS have been seen to improve significantly when treated with TNF inhibitors such as Etanercept, Adalimumab, Infliximab, Certolizumab, and Golimumab [1]. Interleukin-17 blockade using agents such as Bimekizumab and Secukinumab is a novel and efficient therapeutic approach [1].

In an endeavor to ameliorate the current pharmacotherapy, a double-blind, randomized placebo-controlled phase 3 trial (SELECT-AXIS 2) of the drug Upadacitinib, has proven to be significantly efficacious in the management of active non-radiographic axial spondyloarthritis (nr-axSpA), with MRI-based or blood tests displaying objective evidence of inflammation, an increased C-reactive protein, and an unsatisfactory response to Non-steroidal anti-inflammatory drug (NSAIDs) [3]. Immune activation and cytokine production are mediated by the signal transducer and activator of transcription (STAT), which Upadacitinib inhibits selectively in the body by inhibiting Janus-associated kinase (JAK). By the inhibition of this route T-cell activation and the production of pro-inflammatory cytokines which has been shown to improve Rheumatoid Arthritis, Ulcerative Colitis, Psoriatic Arthritis, Atopic dermatitis, and now nr-axSpA [4].

According to research by Deodhar, Atul et al., patients who were using Upadacitinib 15 mg had a highly significant (*p* < 0.0001) ASAS40 (Assessment of SpondyloArthritis International Society 40%) response (45%) at week 14 compared to those receiving a placebo (23%) [3]. This study resulted in the European Commission's approval of Upadacitinib for the treatment of nr-axSpA in the European Union [5].

It is, therefore, crucial to offer an unambiguous treatment choice, especially in light of the disease's strong heredity, its prevalence among the economically active population (the 30s), the lack of oral therapy options, and the stigma associated with surgical intervention. Thus, Upadacitinib's usage in the treatment of nr-axSpA and its clinical trial is a significant step toward the availability of an internationally-approved medicine for the treatment of nr-axSpA. Upadacitinib's efficacy and safety must be evaluated in a much more comprehensive and long-term research, especially among adults who have failed to respond to conventional first and second-line therapy choices for nr-axSpA.

## Ethical approval

Not Applicable.

## Author contribution

Manuscript was written by **MS**, **OHS**, and **GS**. Review editing, formatting and referencing was done by **SO** and **KU**.

## Research registration number

Not Applicable.

## Guarantor

All authors take responsibility for the work, access to data and decision to publish.

## Consent

Not Applicable.

## Financial disclosure

The author(s) received no financial support for the research, authorship, and/or publication of this article.

## Declaration of competing interest

The author(s) declared no potential conflicts of interest concerning the research, authorship, and/or publication of this article.
